# Supplementary material for: Altered microRNA Transcriptome in Cultured Human Liver Cells upon Infection with Ebola Virus
Source: Int J Mol Sci. 2021 Apr 6;22(7):3792. doi: 10.3390/ijms22073792 (PMC8038836; doi:10.3390/ijms22073792)
Supplement: Supplementary file 1 [file ijms-22-03792-s001.zip › Supplementary_File/C_ GO_Analysis_Results/16-30nt_go_Mayinga-96h-Huh7_vs_Control-96h-Huh7_up.mature_mirna_targets/CC_FoldEnrichment.pdf]

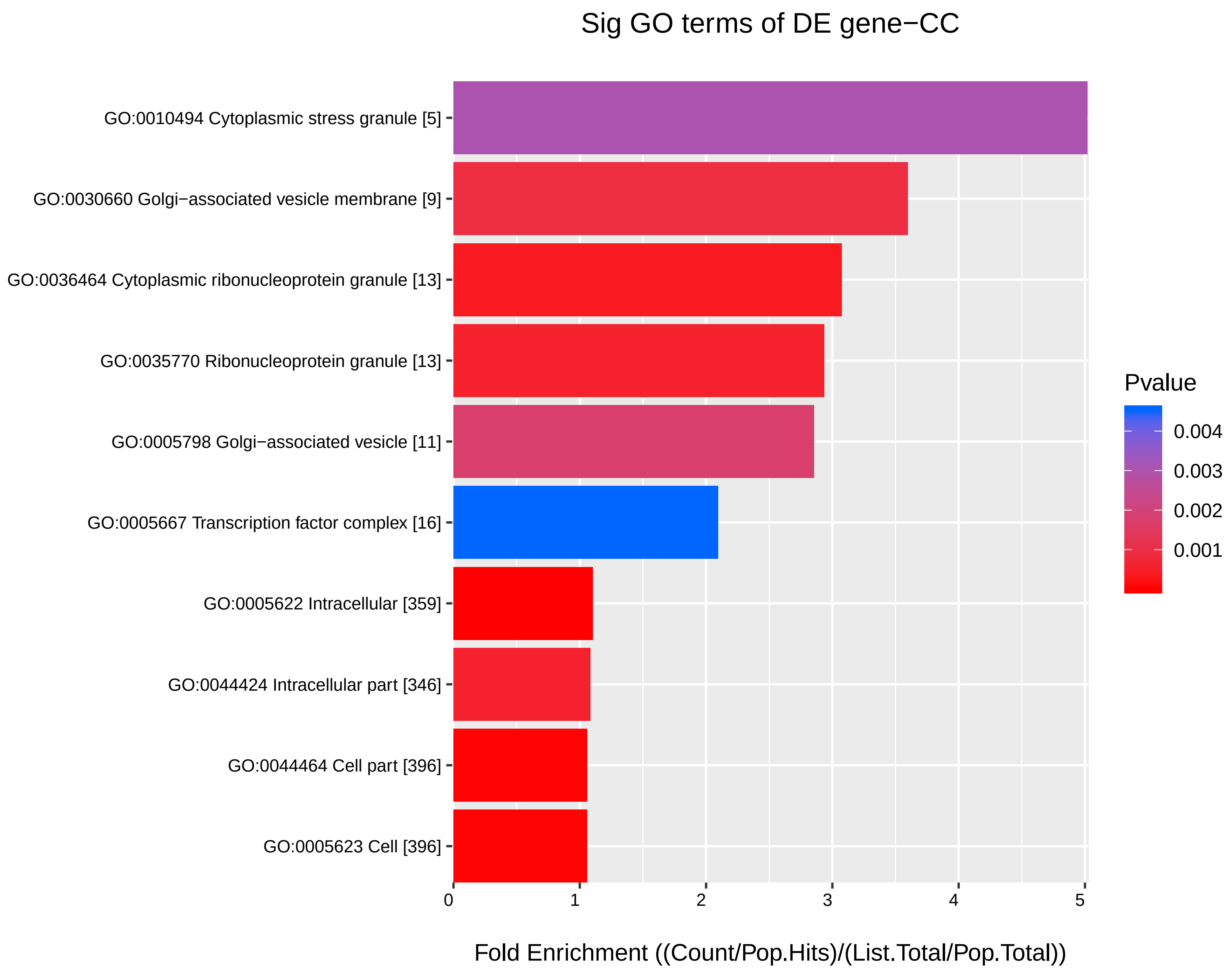
Sig GO terms of DE gene−CC
GO:0010494 Cytoplasmic stress granule [5]
GO:0030660 Golgi−associated vesicle membrane [9]
GO:0036464 Cytoplasmic ribonucleoprotein granule [13]
GO:0035770 Ribonucleoprotein granule [13]
GO:0005798 Golgi−associated vesicle [11]
GO:0005667 Transcription factor complex [16]
GO:0005622 Intracellular [359]
GO:0044424 Intracellular part [346]
GO:0044464 Cell part [396]
GO:0005623 Cell [396]
0
1
2
3
4
5
Fold Enrichment ((Count/Pop.Hits)/(List.Total/Pop.Total))
Pvalue
0.004
0.003
0.002
0.001
